# The Ancient Link between G-Protein-Coupled Receptors and C-Terminal Phospholipid Kinase Domains

**DOI:** 10.1128/mBio.02119-17

**Published:** 2018-01-23

**Authors:** D. Johan van den Hoogen, Harold J. G. Meijer, Michael F. Seidl, Francine Govers

**Affiliations:** a Laboratory of Phytopathology, Wageningen University, Wageningen, The Netherlands; b Business Unit Biointeractions and Plant Health, Wageningen University and Research, Wageningen Plant Research, Wageningen, The Netherlands; Vanderbilt University; Karlsruhe Institute of Technology (KIT)

**Keywords:** G-protein-coupled receptors, *Phytophthora*, cell signaling, oomycetes, phospholipid-mediated signaling

## Abstract

Sensing external signals and transducing these into intracellular responses requires a molecular signaling system that is crucial for every living organism. Two important eukaryotic signal transduction pathways that are often interlinked are G-protein signaling and phospholipid signaling. Heterotrimeric G-protein subunits activated by G-protein-coupled receptors (GPCRs) are typical stimulators of phospholipid signaling enzymes such as phosphatidylinositol phosphate kinases (PIPKs) or phospholipase C (PLC). However, a direct connection between the two pathways likely exists in oomycetes and slime molds, as they possess a unique class of GPCRs that have a PIPK as an accessory domain. In principle, these so-called GPCR-PIPKs have the capacity of perceiving an external signal (via the GPCR domain) that, via PIPK, directly activates downstream phospholipid signaling. Here we reveal the sporadic occurrence of GPCR-PIPKs in all eukaryotic supergroups, except for plants. Notably, all species having GPCR-PIPKs are unicellular microorganisms that favor aquatic environments. Phylogenetic analysis revealed that GPCR-PIPKs are likely ancestral to eukaryotes and significantly expanded in the last common ancestor of oomycetes. In addition to GPCR-PIPKs, we identified five hitherto-unknown classes of GPCRs with accessory domains, four of which are universal players in signal transduction. Similarly to GPCR-PIPKs, this enables a direct coupling between extracellular sensing and downstream signaling. Overall, our findings point to an ancestral signaling system in eukaryotes where GPCR-mediated sensing is directly linked to downstream responses.

## IMPORTANCE

G-protein-coupled receptors (GPCRs) are central sensors that activate eukaryotic signaling and are the primary targets of human drugs. In this report, we provide evidence for the widespread though limited presence of a novel class of GPCRs in a variety of unicellular eukaryotes. These include free-living organisms and organisms that are pathogenic for plants, animals, and humans. The novel GPCRs have a C-terminal phospholipid kinase domain, pointing to a direct link between sensing external signals via GPCRs and downstream intracellular phospholipid signaling. Genes encoding these receptors were likely present in the last common eukaryotic ancestor and were lost during the evolution of higher eukaryotes. We further describe five other types of GPCRs with a catalytic accessory domain, the so-called GPCR-bigrams, four of which may potentially have a role in signaling. These findings shed new light onto signal transduction in microorganisms and provide evidence for alternative eukaryotic signaling pathways.

## INTRODUCTION

To respond to environmental cues and changes, a molecular system that can receive external signals and transduce them to intracellular responses is fundamental for every living organism (1). In eukaryotes, G-protein-coupled receptors (GPCRs) and their associated heterotrimeric G-protein complexes are important and universal signal transduction components that transduce extracellular signals to intracellular signals. A characteristic feature of GPCRs is their topology, with seven transmembrane (TM)-spanning helices flanked by an extracellular N terminus and an intracellular C terminus. Upon binding of a ligand to a GPCR, conformational changes lead to activation and dissociation of the heterotrimeric G-protein complex. G-protein subunits α, β, and γ regulate key effector enzymes such as adenylate cyclase (AC), phospholipase C (PLC), and phosphoinositide 3-kinase, resulting in the production of secondary messengers, such as cAMP, Ca^2+^, and inositol trisphosphate (2). GPCRs are encoded by an evolutionarily ancient gene family that was already present in the last eukaryotic common ancestor (LECA) (1). In mammalians, this is the largest superfamily with genes encoding a very diverse group of transmembrane (TM) proteins. Humans, for example, have over 800 GPCRs (3). They function in a wide variety of processes not only as sensory receptors for taste, smell, or light (rhodopsin) but also as receptors of neurotransmitters, hormones, and nucleotides (3, 4). Consequently, several GPCRs are important targets of modern drugs in human medicine, with over a third of all drugs targeting GPCRs (5). Lower eukaryotes typically have fewer GPCRs than mammalians. For example, the slime mold *Dictyostelium discoideum* has 55 GPCR genes (6) whereas the yeast *Saccharomyces cerevisiae* has only 3 GPCR genes (7, 8).

Our research focuses on plant-pathogenic microbes, organisms for which cellular signaling is of utmost importance for responding to environmental cues but especially for recognizing suitable hosts for infection. Arguably, among the most important groups of plant pathogens are the oomycetes (also known as water molds). Oomycetes occupy environmental niches similar to those occupied by fungi but do not belong to the fungal kingdom (9). Instead, oomycetes belong to the stramenopiles, a lineage that also includes, among others, brown algae and diatoms (10). One of the most notorious oomycetes is the late blight pathogen *Phytophthora infestans*, the culprit responsible for the Irish potato famine in the mid-19th century (11).

Genome comparisons between oomycetes and other eukaryotes revealed distinct features of their gene and protein repertoires (12, 13). The number of distinct protein domain combinations, or bigrams, is significantly higher in oomycetes than in organisms with similar numbers of domain types, and many proteins involved in cellular signaling are composed of unique bigrams (14). For example, there are many oomycete-specific classes of protein kinases with accessory domain combinations not observed in other lineages (15). Another unique protein domain combination was found in the family of GPCR-PIPKs, phosphatidylinositol phosphate kinases (PIPKs) with an N-terminal GPCR (16). The combination of a GPCR and a PIPK domain in one protein suggests that these proteins can function as a link between two important signaling networks, i.e., the G-protein signaling and phospholipid signaling networks. The products of PIPKs, such as PI(4,5)P_2_, are both important membrane components and universal signaling components. For some time, GPCR-PIPKs were thought to have limited phyletic distribution, as only one homologue was identified outside the oomycetes, namely, RpkA (17), a receptor in the slime mold *Dictyostelium discoideum* that is involved in phagocytosis (18). More recently, GPCR-PIPK genes were identified in ciliates and in lower unikonts (19), suggesting that GPCR-PIPKs are more widespread than previously thought.

The aim of this study was to provide a comprehensive overview of the distribution of GPCR-PIPKs among eukaryotes and to gain insight into their origin and evolution. We identified GPCR-PIPKs throughout eukaryotes and reconstructed their molecular phylogeny. With the aim to identify additional GPCR-bigrams, i.e., proteins with a GPCR domain and an accessory domain, we mined the *P. infestans* genome for GPCRs and identified five hitherto-unknown GPCR-bigram types, some of which are shared in other eukaryotes. Of these, four link GPCRs to accessory domains with roles in signaling. Taken together, our results suggest the presence of alternative signaling pathways in eukaryotes, with GPCR-bigrams as central elements.

## RESULTS

### GPCR-PIPKs are conserved in oomycetes.

Previously, GPCR-PIPKs were identified in *Phytophthora* species but also in a few closely related downy mildews, such as *Hyaloperonospora arabidopsidis* and *Plasmopara halstedii* (16, 20–22). Here, we analyzed the genomes of 20 oomycetes (see Table S1 in the supplemental material) for the presence of GPCR-PIPKs. These 20 include species from different genera and are pathogenic for both plants and animals. We found that the GPCR-PIPK family is conserved in all 20 oomycetes, with 9 to 13 GPCR-PIPK genes present in each species (Table S1). The genes were named D1 through D12, in line with the nomenclature of and similarity to *P. infestans* GPCR-PIPKs (Table S2a) (21). Despite the high conservation of the GPCR-PIPK family throughout oomycetes, we observed some differences between species. For example, duplications of several GPCR-PIPKs are found in *Albugo*, *Aphanomyces*, and *Saprolgenia* species, while GPCR-PIPK D9 is found only in *Hyaloperonospora*, *Phytophthora*, and *Pythium* species (Table S2a).

10.1128/mBio.02119-17.4 TABLE S1  Overview of the number of GPCR-bigrams in all species included in this study and references to genome papers. Download TABLE S1, DOCX file, 0.1 MB. Copyright © 2018 van den Hoogen et al. 2018 van den Hoogen et al. This content is distributed under the terms of the Creative Commons Attribution 4.0 International license.

10.1128/mBio.02119-17.5 TABLE S2  Overview of PIPKs. (a) Details of oomycete GPCR-PIPKs. (b) Details of non-oomycete GPCR-PIPKs. (c) Details of oomycete type I, II, and III PIPKs. (d) Details of type I, II, and III PIPKs in species having one or more GPCR-PIPKs. Download TABLE S2, XLSX file, 0.2 MB. Copyright © 2018 van den Hoogen et al. 2018 van den Hoogen et al. This content is distributed under the terms of the Creative Commons Attribution 4.0 International license.

### GPCR-PIPKs are present in various eukaryotic genera.

**FIG 1:**
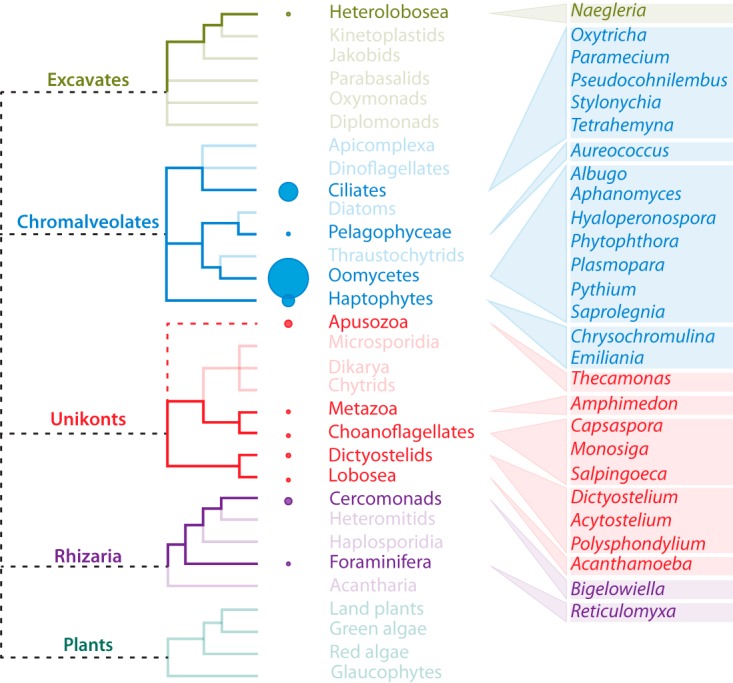
Consensus cladogram of selected eukaryotes, with lineages and genera with species having one or more GPCR-PIPKs highlighted in bold. The size of the circles is proportional to the average number of GPCR-PIPKs per taxon. The tree is based on phylogenies described by Keeling et al. and Koonin (67, 68), and the placement of the *Apusozoa* is based on data reported by Paps et al. (69). Dashed polytomies indicate unresolved relationships.

The finding that GPCR-PIPKs are not limited to oomycetes and amoebozoans but also occur in ciliates and choanoflagellates (18, 19) raised the issue of how widespread GPCR-PIPKs are in eukaryotes. We performed a combination of iterative BLAST, hidden Markov model (HMM), and text-based searches (see Fig. S1a in the supplemental material) against proteomic data from a wide range of species, including archaea, bacteria, and all eukaryotic supergroups. We identified 60 proteins that contain a 7TM domain preceding a PIPK domain and that are thus bona fide GPCR-PIPKs (Table S2b). They are distributed over distantly related taxa in eukaryotic supergroups (Fig. 1), with the exception of the plant kingdom. Within stramenopiles, the lineage that comprises the oomycetes, we could detect GPCR-PIPKs in only one non-oomycete species, the alga *Aureococcus anophagefferens* (Table S1). GPCR-PIPKs occur in several chromalveolates, representing the supergroup that includes the stramenopiles and ciliates. In several ciliates, for example, in *Stylonychia lemnae*, we found numerous GPCR-PIPKs (Table S1). The haptophytes *Emiliania huxleyi* and *Chrysochromulina tobin* possess three and four GPCR-PIPKs, respectively. Haptophytes are a group of marine protists whose origin dates back to approximately 1,000 to 600 million years ago (mya) (23). In unikonts, the supergroup that includes the metazoans, homologues of *D. discoideum* RpkA could be identified in numerous amoebozoans (Table S1). GPCR-PIPKs were also identified in early branching unikonts such as the choanoflagellate *Salpingoeca rosetta* and the *Apusozoa* species *Thecamonas trahens* (Table S1). Additionally, we identified GPCR-PIPKs in other premetazoans such as *Capsaspora owczarzaki* and the sponge *Amphimedon queenslandica*. However, GPCR-PIPKs seem to be absent from multicellular metazoans and fungi. Furthermore, homologues could be found in two *Naegleria* species in the supergroup *Excavates*, and as detailed below, in two species in the supergroup *Rhizaria* (Table S1). *Naegleria* spp. are free-living protists belonging to the *Heterolobosea*, a lineage that diverged from other eukaryotes over a billion years ago (24). In summary, GPCR-PIPKs seem to occur in multiple diverse genera throughout the eukaryotic tree of life and yet they are restricted to unicellular species and, in most cases, to species that diverged early (Fig. 1).

10.1128/mBio.02119-17.1 FIG S1  (A) Search strategy used to identify GPCR-PIPKs. The total numbers of identified GPCR-PIPKs are highlighted in bold. (B) Search strategy used to inventory GPCRs and GPCR-bigrams in *P. infestans*. The numbers in bold represent the number of protein models present after each successive step. Download FIG S1, PDF file, 0.4 MB. Copyright © 2018 van den Hoogen et al. 2018 van den Hoogen et al. This content is distributed under the terms of the Creative Commons Attribution 4.0 International license.

### Most GPCR-PIPKs share a novel central conserved motif, LRxGI.

**FIG 2:**
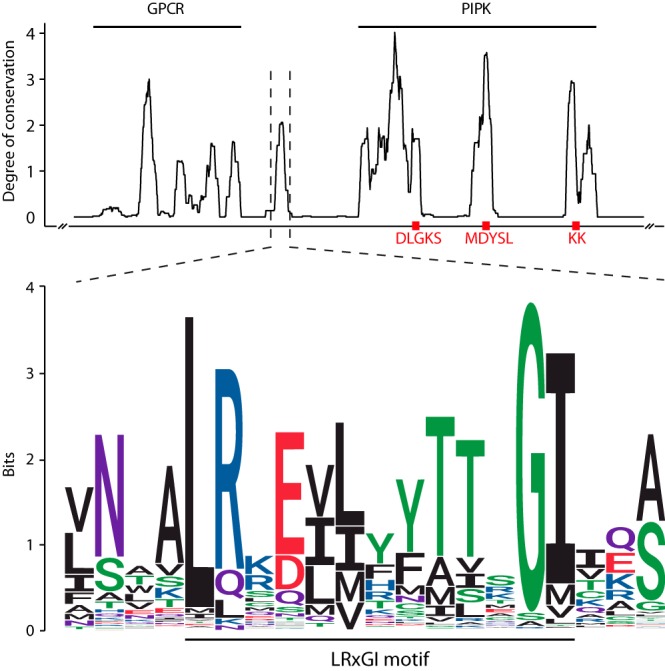
Degree of conservation of all GPCR-PIPKs included in this study (top) and sequence logo of the newly identified LRxGI motif (bottom). The degree of conservation was determined by the use of a sliding moving average over 50 positions. The approximate positions of three conserved PIPK motifs (DLGKS, MDYSL, and KK) are indicated. Color coding of amino acid residues in the sequence logo is shown according to chemical properties as follows: red, acidic; blue, basic; black, hydrophobic; purple, neutral; green, polar.

To identify conserved amino acid motifs in GPCR-PIPKs, we made a multiple-sequence alignment of all identified GPCR-PIPKs. In most GPCR-PIPKs, conserved motifs, such as the catalytic DLGKS and MDYSL motifs (25) and the (di)lysine motif in the activation loop (17), which is involved in substrate specificity (26), can be identified in the PIPK domain (Fig. 2). Between the GPCR and PIPK domain, we observed a short region that is highly conserved (Fig. 2). The highest identity is traced across 13 amino acids, and the consensus sequence starts with a highly conserved leucine-arginine (LR) dimer followed by nine less extensively conserved amino acids and a glycine-isoleucine (GI) dimer at the end (Fig. 2)—further referred to as the LRxGI motif. It consists of both hydrophobic and hydrophilic amino acids, and, according to a preliminary secondary structure prediction using Phyre2 (27), it most likely adopts an α-helical structure (data not shown). The LRxGI motif is present in all 223 oomycete GPCR-PIPKs included in this study. Among the 60 non-oomycete GPCR-PIPKs, the motif is absent in 9 gene models, mostly in ciliate GPCR-PIPKs (Table S2b).

We next examined whether the LRxGI motif is unique for GPCR-PIPKs or also occurs in other protein sequences. Using the motif sequence as a query, BLAST and HMM searches resulted exclusively in hits in GPCR-PIPKs. Notably, two new GPCR-PIPKs were identified that were not detected in the search strategy mentioned above. Hits in *Reticulomyxa filosa*, a species in the supergroup *Rhizaria*, targeted two truncated gene models, one containing a GPCR region N-terminal of the LRxGI motif and the other with a PIPK domain at the C terminus. The genome assembly of *R. filosa* is highly fragmented (28), so the two gene models possibly represent only one GPCR-PIPK gene. Although the sequence overlap of the two gene models is too small, the presence of an LRxGI motif, as a hallmark of a GPCR-PIPK, led us to conclude that *R. filosa* has one or possibly more GPCR-PIPKs. By using the sequence of reconstituted *R. filosa* GPCR-PIPK as the search query, two more homologues in the *Rhizaria* supergroup were identified, namely, in the species *Bigelowiella natans*.

### GPCR-PIPKs have distinct kinase domains.

**FIG 3:**
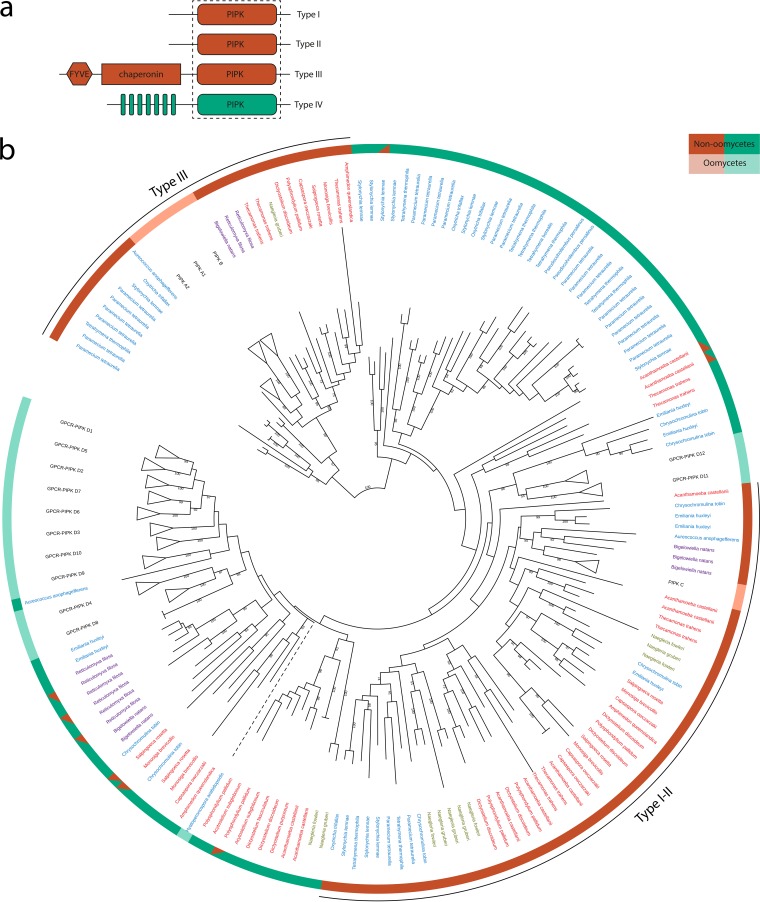
(a) Typical domain organization in the four PIPK types. The regions contained in the dashed box were used for constructing the tree. Protein and domain lengths are not to scale. (b) Phylogenetic tree of type I, II, III, and IV PIPKs. The outer circle shows the classification of PIPKs based on domain composition, with orange representing type I, II, and III PIPKs and lime type IV PIPKs (i.e., GPCR-PIPKs). Split-color bars indicate truncated gene models. The color coding of the species names corresponds to the coding of the supergroup colors described for Fig. 1. The numbers at the nodes represent bootstrap support percentages from RAxML (500 replicates); bootstrap values of <50 are omitted. Clades encompassing oomycete proteins are collapsed and classified based on homology with *P. infestans* PIPK classes (16). Dashed lines are truncated for simplification and indicate arbitrary branch lengths. A tree with noncollapsed clades that includes codes of the gene models is provided in Fig. S2.

We next examined the evolutionary relationship of the GPCR-PIPKs. To this end, we retrieved sequences of all PIPKs that are present in organisms in which we found one or more GPCR-PIPKs (Table S2c and d). A typical eukaryote contains three types of PIPKs, referred to as types I, II, and III, which are grouped based on their catalytic activity and differ in domain composition (Fig. 3a). Since the *Phytophthora* GPCR-PIPKs and *Dictyostelium* RpkA form a separate branch in the phylogenetic tree, they were classified as type IV PIPKs (17). To reconstruct the overall PIPK phylogeny, we included sequences of all four types and used only the regions covering the PIPK domain. Our phylogenetic analyses confirmed earlier studies (16, 17), showing that the catalytic PIPK domains of GPCR-PIPKs (type IV) are distinct from those of the three other PIPK types. Moreover, type IV PIPKs seem to be more closely related to type I and II PIPKs than to type III PIPKs (Fig. 3b). Of the 445 PIPKs present in the tree, 9 are derived from gene models which either are truncated or have equivocal protein domain predictions (Table S2d), and hence the classification based on domain composition is ambiguous. Although classified as type I, II, or III PIPKs, they are most likely type IV PIPKs, i.e., GPCR-PIPKs (Fig. 3b). The relationships between the PIPK domains of oomycete GPCR-PIPKs (Fig. S2) reflect the oomycete species phylogeny (Fig. S3), and as such, it is likely that GPCR-PIPKs and the subsequent expansion of the family are ancestral to oomycetes. Group D11 and D12 GPCR-PIPKs form a clade separate from other GPCR-PIPKs (Fig. 3b), which might indicate a separate origin.

10.1128/mBio.02119-17.2 FIG S2  Phylogenetic tree of type I, II, III, and IV PIPKs. The outer circle shows the classification of PIPKs based on domain composition, with orange representing type I, II, and III PIPKs and lime type IV PIPKs (i.e., GPCR-PIPKs). Split-color bars indicate truncated gene models. The color coding of the species names corresponds to the coding of the supergroup colors described for Fig. 1. The numbers at the nodes represent bootstrap support percentages from RAxML (500 replicates). Bootstrap values of <50 are omitted. Download FIG S2, PDF file, 21.2 MB. Copyright © 2018 van den Hoogen et al. 2018 van den Hoogen et al. This content is distributed under the terms of the Creative Commons Attribution 4.0 International license.

10.1128/mBio.02119-17.3 FIG S3  Simplified phylogeny of oomycetes and related organisms based on the supertree as presented by McCarthy et al. (70). Download FIG S3, PDF file, 0.1 MB. Copyright © 2018 van den Hoogen et al. 2018 van den Hoogen et al. This content is distributed under the terms of the Creative Commons Attribution 4.0 International license.

Some GPCR-PIPKs in phytoplanktonic species are present within the clades comprising primarily oomycete GPCR-PIPKs. For example, an *A. anophagefferens* GPCR-PIPK forms a clade together with the oomycete GPCR-PIPK D9 (Fig. 3b). The placement of two *E. huxleyi* and two *C. tobin* GPCR-PIPKs in the clade containing oomycete GPCR-PIPKs D11 and D12 is strongly supported by bootstrap analysis (Fig. 3b). This indicates that an ancestral GPCR-PIPK was present in the ancestor of oomycetes, haptophytes, and *Pelagophyceae*, which are three lineages within the chromalveolates. Notably, all ciliate GPCR-PIPKs cluster in one clade. Even though their phylogenetic position might suggest that these GPCR-PIPKs arose independently of the other GPCR-PIPKs, the phylogenetic support is insufficient to firmly establish this hypothesis. The recent whole-genome duplication events in *P. tetraurelia* (29) are clearly reflected in duplications of GPCR-PIPKs. A similar pattern is observed in the other ciliates included in this study.

### Novel types of GPCR-bigrams are present in oomycetes.

Oomycetes have an expanded repertoire of bigrams (14), and many of these unique bigrams are predicted to have a role in cellular signaling. GPCR-PIPKs are one example, but it is possible that there are other GPCR-bigrams that have the ability to link GPCR-mediated sensing directly to specific downstream signaling pathways. To identify other GPCR-bigrams, we first set out to identify all GPCRs present in *P. infestans* and then selected the subset in which the GPCR domain is flanked by an accessory domain. These were then used as a query to search for homologues in other oomycetes and, more widely, in other eukaryotes.

**FIG 4:**
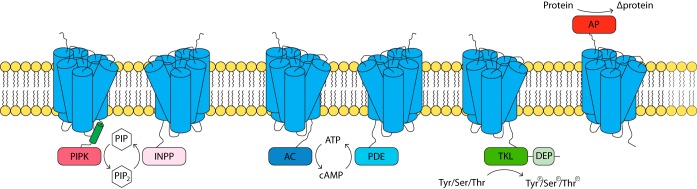
Schematic representation of the domain organization of GPCR-bigrams in oomycetes and their predicted catalytic activities. Blue cylinders represent transmembrane helices. PIPK, phosphatidylinositol-4-phosphate 5-kinase; INPP, inositol polyphosphate phosphatase; PIP, phosphatidylinositol; AC, adenylyl cyclase; PDE, phosphodiesterase; DEP, Dishevelled, Egl-10, and pleckstrin; AP, aspartic protease.

Identification of GPCRs based on protein sequence can be troublesome due to the limited sequence conservation across phylogenetic lineages. Hence, we exploited the most characteristic attribute of GPCRs, their membrane topology, with seven TM-spanning domains. To identify putative GPCRs in *P. infestans*, we scanned the proteome for TM domains (Fig. S1b). Approximately 18% of the gene models were predicted to encode TM-containing proteins, which is comparable to the proportion seen with other eukaryotes (data not shown). From a total of approximately 3,200 gene models, we selected 687 with 5 to 9 predicted TM regions. We then used different tools to predict accessory domains and discarded those encoding known non-GPCR TM proteins, such as transporters and ion channels. This resulted in 132 candidates with a putative GPCR signature (Table S3a). Of these, 44 were GPCRs with an N- or C-terminal catalytic accessory domain, or GPCR-bigrams. These included the GPCR-PIPKs, but in addition, five novel types of GPCR-bigrams were identified (Fig. 4) and are described below. Homologues for each of these GPCR-bigrams were found in other oomycetes, and the copy numbers in each species were in the same range as in *P. infestans*. However, the distribution of the other GPCR-bigrams outside the oomycetes was more restricted than the distribution of GPCR-PIPKs.

10.1128/mBio.02119-17.6 TABLE S3  Overview of GPCRs in *P. infestans* and GPCR-bigrams in eukaryotes. (a) Inventory of GPCRs and GPCR-bigrams in *P. infestans*. (b) Details of GPCR-TKLs. (c) Details of GPCR-INPPs. (d) Details of GPCR-AC. (e) Details of GPCR-PDEs. (f) Details of AP-GPCRs. Download TABLE S3, XLSX file, 0.3 MB. Copyright © 2018 van den Hoogen et al. 2018 van den Hoogen et al. This content is distributed under the terms of the Creative Commons Attribution 4.0 International license.

Of the 44 GPCR-bigrams found to be encoded on the *P. infestans* genome, 17 have a C-terminal tyrosine kinase-like (TKL) domain, making the GPCR-TKLs the largest group of GPCR-bigrams in *P. infestans*. The occurrence of bigrams consisting of a TKL domain and TM domains was already reported previously in a kinome inventory of *P. infestans* (15), but the notion that these proteins have a GPCR signature was not mentioned. Of the 17 *P. infestans* GPCR-TKLs, 8 have a single DEP (Dishevelled, Egl-10, and pleckstrin) domain located C-terminally of the kinase domain (Table S3b). The DEP domain is found in several proteins involved in G-protein signaling and is important for interactions of those proteins with various partners at the membrane, including phospholipids and membrane receptors (71).

The number of GPCR-TKL genes in most oomycetes is similar to that in *P. infestans* (Table S1). *H. arabidopsidis*, however, has only four homologues (Table S1), which is in line with previous reports that the kinome of *H. arabidopsidis* is smaller than that of *P. infestans* (15). The low number of GPCR-TKL genes predicted in *P. capsici* is likely due to poor coverage and/or annotation of the genome sequence. The slime mold *D. discoideum* has two proteins resembling oomycete GPCR-TKLs but lacking the DEP domain (31), with homologues in other amoebozoans (Table S1). We have no evidence for the presence of GPCR-TKLs in organisms from taxa other than oomycetes or amoebozoans.

The third largest group after the GPCR-TKLs and GPCR-PIPKs comprises GPCR-bigrams that have an inositol polyphosphate phosphatase (INPP) domain as an accessory domain. *P. infestans* has seven GPCR-INPP genes, and between one and nine homologues are present in other oomycetes (Table S1). INPPs are phosphatases that can dephosphorylate PIP_2_. Since PIP_2_ is a product of PIPK activity, we speculate that the GPCR-PIPKs and GPCR-INPPs in oomycetes operate as a couple in a cycle of phospholipid phosphorylation-dephosporylation. In other eukaryotic taxa, the presence of GPCR-INPPs is limited to the microalga *Nannochloropsis gaditana*, a species that is in the stramenopile lineage and thus is relatively closely related to oomycetes. Like *P. infestans*, *N. gaditana* has seven GPCR-INPP genes, but in contrast, it lacks GPCR-PIPKs (Table S1).

Two novel types of GPCR-bigrams identified in this study are likely involved in cAMP signaling. One type, encoded by four *P. infestans* gene models, has adenylyl cyclase (AC) as a C-terminal accessory domain, while the other type, encoded by three gene models, has phosphodiesterase (PDE) as a C-terminal accessory domain. Adenylyl cyclases catalyze the conversion of ATP into cyclic AMP, while PDE can hydrolyze cAMP or other cyclic nucleotides to ATP or NTP, respectively. Other oomycetes have comparable numbers of genes encoding GPCR-ACs and GPCR-PDEs, although genes encoding GPCR-PDEs could not be identified in some species (Table S1). Outside oomycetes, no homologues of GPCR-ACs or GPCR-PDEs were found. These findings imply that, like phospholipid signaling, cAMP signaling in oomycetes is to some extent regulated by proteins with a N-terminal GPCR domain.

The last of the six types of GPCR-bigrams is an outlier. This GPCR-bigram has a C-terminal GPCR domain instead of an N-terminal GPCR domain, while the N terminus harbors aspartic protease (AP) as an accessory domain. *P. infestans* has one AP-GPCR gene, *PiAP5*, which is one of the 12 *P. infestans* genes encoding an AP (32). On the basis of the predicted membrane topology of AP5, we assume that the catalytic domain is located extracellularly. This assumption is supported by the presence of a signal peptide, which is likely required for proper translocation of the AP domain across the endoplasmic reticulum membrane during posttranslational processing of the protein. All other oomycetes have a single homologue of *PiAP5* (Table S1). Outside the oomycetes, however, no proteins with similarity to AP-GPCR could be identified.

## DISCUSSION

Domain rearrangements are perhaps among the most important factors in the evolution of multidomain proteins (33). Such mechanisms, by recombining existing domains, can lead to the creation of novel multidomain proteins, possibly including proteins with different or new functions. Many multidomain proteins, often with domain combinations not found elsewhere, have been observed previously in oomycetes (14). Several of these proteins have a GPCR domain, indicating a direct link between environmental sensing and intracellular signaling (16). Here we provide evidence for a more widespread presence of one such bigram type, the GPCR-PIPK. Also, we show that, in addition to GPCR-PIPKs, oomycetes have five other distinct types of GPCR-bigrams, four of which have potential roles in signal transduction.

GPCR-PIPKs were first identified in the genus *Phytophthora* (16). In this report, we show that all oomycetes species that have been sequenced to date have a family of at least nine GPCR-PIPKs and that several species in taxa other than oomycetes also have GPCR-PIPKs, albeit typically in smaller numbers. We identified GPCR-PIPKs in species belonging to four of the five eukaryotic supergroups, with the exception being plants; however, their presence is limited to a relatively small number of organisms. These organisms are evolutionarily very distantly related. For example, the evolutionary distance between the genera *Phytophthora* and *Naegleria* is extremely large and is likely similar to the distance to the LECA, i.e., over a billion years (30, 34). Given that horizontal gene transfer (HGT) events are rare (35) and that the general species phylogeny is reflected in the phylogenetic reconstruction of GPCR-PIPKs, HGT is unlikely to have had a major role in the evolution of GPCR-PIPKs. Moreover, features shared by *Naegleria* spp. and other eukaryotic groups are likely to have existed in their common ancestor (24). Consequently, the widespread presence of GPCR-PIPKs in taxonomically unrelated groups is best explained by a shared ancestry in the LECA, fitting earlier hypotheses (1).

Despite their evolutionary distance, organisms with GPCR-PIPKs share some features. The first, and most obvious, is that all can be regarded as unicellular microorganisms. Second, most organisms occupy similar environmental niches, residing in a watery environment, and have an important swimming life stage. While some are harmless microorganisms, many species, such as the human pathogens *Naegleria fowleri* and *Acanthamoeba castellanii* and the many plant and animal pathogenic oomycetes, are important pathogens. Others, such as *Aureococcus anophagefferens* and *Emiliania huxleyi*, have an important environmental impact and can cause harmful algal blooms.

Although GPCRs and G-proteins are ubiquitously present in metazoans, GPCR-bigrams are rare or even nonexistent. Some metazoan GPCRs contain N- or C-terminal extensions, but these do not contain catalytic domains. Since GPCR-PIPK genes can be identified in the common ancestors of metazoans, such as sponges and choanoflagellates, it is likely that these genes were lost during the expansive evolution of the G-protein signaling network in higher metazoans. Notably, GPCR-PIPKs are absent in plants, including lower plants such as red algae and glaucophytes. However, GPCRs are also rare in plants, and plant G-proteins are self-activating and might act independently of GPCRs (36). Only one putative GPCR has been described in *Arabidopsis thaliana* (37), but its precise role remains to be determined. It is possible that the ancestral GPCR-PIPK was lost together with other GPCRs during the early evolution of plants.

Our searches covered a wide variety of species, including archaea, bacteria, and organisms from all eukaryotic supergroups. With the vast number of high-quality genome sequences of higher metazoans and plants available nowadays, it is unlikely that we have failed to detect GPCR-PIPKs in these highly developed multicellular organisms, so we are confident in stating that these organisms do not have GPCR-PIPKs. In contrast, this is less certain for other lineages, such as diatoms, brown algae, *Rhizaria*, and *Excavates*, from which only a limited number of genomes have been sequenced. Consequently, it is not unlikely that, with the release of new or higher-quality genome sequences, more species with GPCR-PIPKs can and likely will be identified.

We have not observed a correlation between the presence of GPCR-PIPKs and the overall number of GPCRs, heterotrimeric G-proteins, or GPCR-regulatory proteins. For example, whereas some organisms identified to have GPCR-PIPKs have no or only a very limited number of Gα subunits, *N. gruberi* and *B. natans* have over 30 Gα subunits (1). Similarly, 229 regulators of G-protein signaling (RGS) genes are present in the *N. gruberi* genome, while a very limited number is present in other organisms with GPCR-PIPKs (1).

The LRxGI motif is strictly limited to GPCR-PIPKs and is present in nearly all GPCR-PIPKs included in this study. The presence of such a specific and unique motif further strengthens the hypothesis of a shared ancestry of GPCR-PIPKs. The LRxGI motif is located relatively close to the GPCR region, leading to the inference that it is not likely to be an integral part of the PIPK domain. Its amphilicity, predicted secondary structure, and proximity to the 7TM region are reminiscent of the characteristics of a structural motif that is often found in mammalian rhodopsin-like GPCRs as well as in angiotensin type 1 receptor (AT1R) (38). This amphipathic “helix 8” (H8) is present just following the last TM domain and lies parallel to the cytoplasmic membrane surface. H8 has been shown to be involved in G-protein activation, conformational stabilization of the GPCR, and phospholipid interactions (38–40). For example, H8 of AT1R specifically interacts with PI(4)P (38). By disrupting the structural organization of the membrane, this interaction might trigger a conformational change of the GPCR (38). Hypothetically, the LRxGI motif could operate by a similar mechanism. By forcing itself into the plasma membrane, potentially directed by PI(4)P, it could aid in phosphorylation by bringing the PIPK domain into closer proximity to the membrane. Alternatively, the LRxGI motif could serve as a linker to connect the GPCR and PIPK domain and modulate (a) conformational shift(s). This is not very likely, however, as this would not explain the particularly high sequence conservation in a relatively small region of the sequence linking the GPCR and PIPK domain.

While GPCR-PIPKs are present throughout the eukaryotic kingdom, the distribution of other GPCR-bigrams is more limited. Apart from oomycetes, GPCR-TKLs are found only in *Amoebozoa* and GPCR-INPPs are found only in the stramenopile alga *Nannochloropsis*, while GPCR-ACs, GPCR-PDEs, and AP-GPCR are restricted to oomycetes. The large number of genes encoding GPCR-bigrams in oomycetes (around 44 in each species) strongly suggests that these organisms evolved toward G-protein signaling pathways. The catalytic domains found in GPCR-bigrams are also found in proteins with a canonical domain organization. For example, *P. infestans* has four GPCR-ACs in addition to eight ACs with a universal domain composition (data not shown).

The processes in which GPCR-bigrams are predicted to be involved are typically regulated by G-proteins. For example, many ACs are activated by stimulatory Gα subunits (G_s_α) or Gβγ subunits and are deactivated by inhibitory Gα subunits (G_i_α). Higher metazoans have multiple Gα, Gβ, and Gγ subunits. Combinations of different subunits give rise to a large variety of heterotrimeric G-protein complexes, each with specific functions. In contrast, oomycetes have only one Gα, Gβ, and Gγ subunit and thus only one uniform heterotrimeric G-protein complex. Silencing of the Gα subunit gene or the Gβ subunit gene in *P. infestans* results in aberrant swimming behavior of zoospores or deficiencies in sporulation, respectively, but does not affect viability (41, 42). This shows that the heterotrimeric G-protein complex is not by definition the preferred partner of GPCRs in all life stages of *P. infestans*. With respect to regulating AC activity, GPCR-ACs and GPCR-PDEs might provide a direct link between GPCR sensing and the cAMP signal transduction pathway, thereby bypassing G-protein intermediates.

Similarly, the large group of GPCR-TKLs might harness oomycetes with an alternative GPCR desensitization pathway. Desensitization of ligand-activated GPCRs is typically initiated by recruitment of β-arrestins upon phosphorylation of residues within the intracellular loop and C-terminal tail of the GPCRs. The kinases involved are G-protein-coupled receptor kinases (GRKs), cAMP-dependent protein kinase A (PKA), or protein kinase C (PKC) (43). However, only one GRK was detected in *P. infestans* and no PKC was detected (15). PKC is also absent from other chromalveolates, such as *Plasmodium*, and from *Arabidopsis* (plants), *Giardia* (*Excavates*), and *Dictyostelium* (unikonts) (31, 44). It is present in yeast and higher unikonts, though, and it has hence been suggested that it arose late in evolution (31). GPCR-TKLs possibly function as substitutes for PKC. Nearly half of the oomycete GPCR-TKLs contain a C-terminal DEP domain, which is a domain also found in some RGS proteins that interact with internal loop regions and the intracellular C-terminal tails of GPCRs via the DEP domain (45). In *S. cerevisiae*, for example, the DEP domain has been shown to be involved in desensitization of GPCR signaling responses (46). The presence of a DEP domain in GPCR-TKLs further suggests a function in regulation of G-protein signaling.

With respect to phospholipid signaling, the presence of GPCR-PIPKs and GPCR-INPPs is indicative of alternative pathways in oomycetes. Among the typical activators of PIPKs are Rho GTPases (47), PKA (48), and phosphatidic acid (48). In *P. infestans*, only two proteins have similarity to Rho GTPases. Whether GPCR-PIPKs and GPCR-INPPs indeed bypass G-protein or other signaling intermediates by directly activating the catalytic domain upon ligand binding remains to be tested experimentally.

Thus far, experimental data on GPCR-bigrams are very limited and hence their function and catalytic activity remain elusive. Functions have been elucidated for some GPCR-PIPKs, though. Expression profiling showed that all 12 GPCR-PIPKs present in *P. infestans* and *P. sojae* are differentially expressed during development (49), and functional gene analyses based on gene silencing of three GPCR-PIPKs revealed roles in sexual reproduction and virulence (49, 50). In *D. discoideum*, *RpkA* knockout mutants showed defects in bacterial defense and had reduced levels of phosphoinositides (18). However, nothing is known with respect to their catalytic activity, and the same holds true for the other GPCR-bigrams. For example, we cannot exclude the possibility that one or several of the predicted AC or PDE domains might actually function as a guanylyl cyclase or a cGMP phosphodiesterase, respectively. Similarly, even though the kinase domains of GPCR-TKLs have sequence similarity to tyrosine kinases, they might well act biochemically as serine/threonine kinases (15, 31). Elucidating the function and catalytic activity of these peculiar GPCR-bigrams and their roles in sensing and signaling might be instrumental for identifying novel classes of drug targets to combat pathogenic microorganisms.

### Conclusion.

The presence of six distinct types of GPCR-bigrams in oomycetes strongly suggests the presence of alternative G-protein signaling pathways. While a wide distribution of GPCR-PIPKs is observed throughout the eukaryotic kingdom, other GPCR-bigrams are more restricted in their presence. On the basis of our findings, it is most likely that GPCR-PIPKs are evolutionarily ancient and that they were likely already present in LECA, a hypothesis that is further strengthened by the ubiquitous presence of the unique, highly conserved LRxGI motif adjacent to the GPCR domain. Whether and how GPCR-bigrams regulate catalytic activity of the accessory domains remain to be elucidated experimentally. Taking the data together, the discovery of GPCR-bigrams and of the widespread presence of GPCR-PIPKs reveals an additional layer of the already intricate G-protein signaling pathway in eukaryotes and points to the presence of novel signaling pathways in microorganisms.

## MATERIALS AND METHODS

### Search strategy for identification of GPCR-PIPKs.

To identify GPCR-PIPKs, we applied an iterative search strategy (depicted in Fig. S1a in the supplemental material). Protein sequences of previously annotated GPCR-PIPKs (16, 18–20) were used as queries for BLAST searches (*E* value cutoff score of 1 e^−1^) against the NCBI, UniProt, FungiDB (51), and EuPathDB databases (52) (last accessed 5 May 2017). These databases cover proteomes of a wide range of species, including archaea, bacteria, and organisms from all eukaryotic supergroups. In parallel, HMM searches (53) (default settings) were performed on the HMMER webserver, using individual protein sequences (phmmer) or multiple-sequence alignments of full-length sequences, the PIPK domain, and the LRxGI motif of GPCR-PIPKs as a query (hmmsearch, jackhmmer). These methods were supplemented by text-based searches in the Uniprot database using keywords and protein domain identifiers. All potential candidates were analyzed for protein domain composition using the SMART database (54) and Pfam, Prints, and TIGRFAMs under InterProScan (55). TM domains were predicted using Phobius (56), TMHMM (57), HMMTOP (58), GPCRHMM (59), and SOSUI (60). Proteins containing domains other than PIPK at the C terminus and those without TM domains were discarded. Newly identified GPCR-PIPKs were then used in queries for iterative searches using the methods described above. All identified GPCR-PIPK sequences are listed in Table S2 in the supplemental material.

The degree of conservation of GPCR-PIPKs was calculated in Jalview using the AMAS method (61), and those data are presented as a sliding moving average calculated over 50 positions. The WebLogo for the LRxGI motif was plotted using the ggseqlogo package (62) in R v3.3.2.

### Retrieval of PIPK genes.

All genomes in which GPCR-PIPK genes were identified (Table S1) were screened for type I, II, and III PIPK genes by BLAST, HMM searches, and text-based searches. All resulting hits were validated by analyzing the predicted protein domain composition using SMART (54) and Pfam, Prints, and TIGRFAMs under InterProScan (55). All identified PIPK genes are listed in Table S2.

### Phylogenetic reconstruction of PIPKs.

PIPK domain regions were extracted from type I, II, III, and IV (GPCR-PIPKs) PIPKs, using the positions of SMART and InterProScan protein domain predictions as a guide (see the positions of PIPK domain regions listed in Table S2). Extracted protein domains were aligned using MAFFT v7 (algorithm: G-INS-i) (63) under Geneious version r9.1.4 (64). After alignment positions that contained more than 25% gaps were trimmed, a phylogenetic tree was reconstructed using the WAG amino acid substitution model with a gamma model of rate heterogeneity, and bootstrapping (500 replicates) was performed with rapid bootstrap analyses on RAxML v8.2.4 (65). The phylogeny was annotated as a phylogram using the Interactive Tree of Life (iTOL) website (66).

### *P. infestans* GPCR inventory.

For making an inventory of *P. infestans* proteins with a GPCR signature, the proteome was screened for proteins with 5 to 9 TM regions using methods described above. Next, the protein domain composition was predicted using methods described above. Protein models with sequence similarity to non-GPCR membrane proteins, such as ion channels, transporters, or non-GPCR receptor proteins, were used. All identified GPCRs and GPCR-bigrams are listed in Table S3.
