# Supplementary material for: The Ancient Link between G-Protein-Coupled Receptors and C-Terminal Phospholipid Kinase Domains
Source: mBio. 2018 Jan 23;9(1):e02119-17. doi: 10.1128/mBio.02119-17 (PMC5784254; doi:10.1128/mBio.02119-17)
Supplement: TABLE S1 [file mbo001183681st1.docx]

Table S1. Overview of number of GPCR-bigrams in all species included in this study, and references to genome papers. Species are grouped according to their phylogenetic position.

| Supergroup | Taxon | Species | GPCR-PIPKs | GPCR-INPPs | GPCR-ACs | GPCR-PDEs | GPCR-TKLs | GPCR-APs | Genome paper |
| --- | --- | --- | --- | --- | --- | --- | --- | --- | --- |
| Excavates | Heterolobosea | *Naegleria fowleri* | 1 |  |  |  |  |  | (1) |
|  |  | *Naegleria gruberi* | 1 |  |  |  |  |  | (2) |
| Chromalveolates | Ciliates | *Oxytricha trifellax* | 2 |  |  |  |  |  | (3) |
|  |  | *Paramecium tetraurelia* | 15 |  |  |  |  |  | (4) |
|  |  | *Tetrahymena thermophila* | 6 |  |  |  |  |  | (5) |
|  |  | *Tetrahymena borealis* | 1 |  |  |  |  |  | ^1^ |
|  |  | *Stylonychia lemnae* | 6 |  |  |  |  |  | (6) |
|  |  | *Pseudocohnilembus persalinus* | 2 |  |  |  |  |  | (7) |
|  | Pelagophyceae | *Nannochloropsis gaditana* |  | 7 |  |  |  |  | (8) |
|  |  | *Aureococcus anophagefferens* | 1 |  |  |  |  |  | (9) |
|  | Oomycetes | *Albugo candida* | 11 | 1 | 2 |  | 6 | 1 | (10) |
|  |  | *Albugo laibachii* | 11 | 1 | 4 | 1 | 8 | 1 | (11) |
|  |  | *Aphanomyces astaci* | 12 | 4 | 3 |  | 13 | 1 | ^1^ |
|  |  | *Aphanomyces invadans* | 11 | 4 | 3 | 2 | 13 | 1 | ^1^ |
|  |  | *Hyaloperonospora arabidopsidis* | 11 | 1 | 3 |  | 4 | 1 | (12) |
|  |  | *Phytophthora capsici* | 11 | 2 | 3 |  | 6 | 1 | (13) |
|  |  | *Phytophthora cinnamomi* | 12 | 4 | 7 |  | 16 | 1 | (14) |
|  |  | *Phytophthora infestans* | 12 | 7 | 4 | 3 | 17 | 1 | (15) |
|  |  | *Phytophthora parasitica* | 12 | 4 | 6 | 2 | 18 | 1 | ^1^ |
|  |  | *Phytophthora ramorum* | 11 | 3 | 7 |  | 13 | 1 | (16) |
|  |  | *Phytophthora sojae* | 12 | 5 | 7 | 2 | 14 | 1 | (16) |
|  |  | *Plasmopara halstedii* | 11 | 6 | 5 |  | 12 | 1 | (17) |
|  |  | *Pythium aphanidermatum* | 9 | 4 | 4 | 2 | 9 | 1 | (18) |
|  |  | *Pythium arrhenomanes* | 9 | 4 | 8 | 2 | 11 | 1 | (18) |
|  |  | *Pythium irregulare* | 11 | 3 | 6 | 2 | 20^2^ | 1 | (18) |
|  |  | *Pythium iwayamai* | 10 | 6 | 2 | 2 | 17^2^ | 1 | (18) |
|  |  | *Pythium ultimum* | 12 | 3 | 8 | 2 | 13 | 1 | (18) |
|  |  | *Pythium vexans* | 9 | 4 | 6 | 2 | 13 | 1 | (18) |
|  |  | *Saprolegnia diclina* | 12 | 6 | 3 | 3 | 16 | 1 | ^1^ |
|  |  | *Saprolegnia parasitica* | 14 | 9 | 1 | 3 | 17 | 1 | (19) |
|  | Haptophytes | *Chrysochromulina tobin* | 4 |  |  |  |  |  | (20) |
|  |  | *Emiliania huxleyi* | 3 |  |  |  |  |  | (21) |
| Unikonts | Apusozoa | *Thecamonas trahens* | 2 |  |  |  |  |  | ^1^ |
|  | Metazoa | *Amphimedon queenslandica* | 1 |  |  |  |  |  | (22) |
|  | Choanoflagellates | *Capsaspora owczarzaki* | 1 |  |  |  |  |  | (23) |
|  |  | *Salpingoeca rosetta* | 1 |  |  |  |  |  | (24) |
|  |  | *Monosiga brevicollis* | 1 |  |  |  |  |  | (25) |
|  | Dictyostelids | *Acytostelium subglobosum* | 2 |  |  |  |  |  | (26) |
|  |  | *Dictyostelium discoideum* | 1 |  |  |  | 2 |  | (27) |
|  |  | *Dictyostelium fasciculatum* | 1 |  |  |  | 1 |  | (28) |
|  |  | *Dictyostelium purpureum* | 1 |  |  |  | 1 |  | (29) |
|  |  | *Polysphondylium pallidum* | 1 |  |  |  | 1 |  | (28) |
|  | Lobosea | *Acanthamoeba castellanii* | 2 |  |  |  | 2 |  | (30) |
| Rhizaria | Foraminifera | *Reticulomyxa filosa* | 1-4^3^ |  |  |  |  |  | (31) |
|  | Cercomonads | *Bigelowiella natans* | 2 |  |  |  |  |  | (32) |

^1^ Broad Institute, no genome paper

^2^ Some partial genes, the actual number of GPCR-TKLs is likely lower

^3^ No full-length GPCR-PIPK genes

**References**

1. Zysset-Burri DC, Muller N, Beuret C, Heller M, Schurch N, Gottstein B, Wittwer M. 2014. Genome-wide identification of pathogenicity factors of the free-living amoeba Naegleria fowleri. BMC Genomics 15:496.

2. Fritz-Laylin LK, Prochnik SE, Ginger ML, Dacks JB, Carpenter ML, Field MC, Kuo A, Paredez A, Chapman J, Pham J, Shu S, Neupane R, Cipriano M, Mancuso J, Tu H, Salamov A, Lindquist E, Shapiro H, Lucas S, Grigoriev IV, Cande WZ, Fulton C, Rokhsar DS, Dawson SC. 2010. The genome of *Naegleria gruberi* illuminates early eukaryotic versatility. Cell 140:631-42.

3. Swart EC, Bracht JR, Magrini V, Minx P, Chen X, Zhou Y, Khurana JS, Goldman AD, Nowacki M, Schotanus K, Jung S, Fulton RS, Ly A, McGrath S, Haub K, Wiggins JL, Storton D, Matese JC, Parsons L, Chang WJ, Bowen MS, Stover NA, Jones TA, Eddy SR, Herrick GA, Doak TG, Wilson RK, Mardis ER, Landweber LF. 2013. The Oxytricha trifallax macronuclear genome: a complex eukaryotic genome with 16,000 tiny chromosomes. PLoS Biol 11:e1001473.

4. Aury JM, Jaillon O, Duret L, Noel B, Jubin C, Porcel BM, Segurens B, Daubin V, Anthouard V, Aiach N, Arnaiz O, Billaut A, Beisson J, Blanc I, Bouhouche K, Camara F, Duharcourt S, Guigo R, Gogendeau D, Katinka M, Keller AM, Kissmehl R, Klotz C, Koll F, Le Mouel A, Lepere G, Malinsky S, Nowacki M, Nowak JK, Plattner H, Poulain J, Ruiz F, Serrano V, Zagulski M, Dessen P, Betermier M, Weissenbach J, Scarpelli C, Schachter V, Sperling L, Meyer E, Cohen J, Wincker P. 2006. Global trends of whole-genome duplications revealed by the ciliate *Paramecium tetraurelia*. Nature 444:171-8.

5. Eisen JA, Coyne RS, Wu M, Wu D, Thiagarajan M, Wortman JR, Badger JH, Ren Q, Amedeo P, Jones KM, Tallon LJ, Delcher AL, Salzberg SL, Silva JC, Haas BJ, Majoros WH, Farzad M, Carlton JM, Smith RK, Jr., Garg J, Pearlman RE, Karrer KM, Sun L, Manning G, Elde NC, Turkewitz AP, Asai DJ, Wilkes DE, Wang Y, Cai H, Collins K, Stewart BA, Lee SR, Wilamowska K, Weinberg Z, Ruzzo WL, Wloga D, Gaertig J, Frankel J, Tsao CC, Gorovsky MA, Keeling PJ, Waller RF, Patron NJ, Cherry JM, Stover NA, Krieger CJ, del Toro C, Ryder HF, Williamson SC, et al. 2006. Macronuclear genome sequence of the ciliate Tetrahymena thermophila, a model eukaryote. PLoS Biol 4:e286.

6. Aeschlimann SH, Jonsson F, Postberg J, Stover NA, Petera RL, Lipps HJ, Nowacki M, Swart EC. 2014. The draft assembly of the radically organized Stylonychia lemnae macronuclear genome. Genome Biol Evol 6:1707-23.

7. Xiong J, Wang G, Cheng J, Tian M, Pan X, Warren A, Jiang C, Yuan D, Miao W. 2015. Genome of the facultative scuticociliatosis pathogen Pseudocohnilembus persalinus provides insight into its virulence through horizontal gene transfer. Sci Rep 5:15470.

8. Radakovits R, Jinkerson RE, Fuerstenberg SI, Tae H, Settlage RE, Boore JL, Posewitz MC. 2012. Draft genome sequence and genetic transformation of the oleaginous alga Nannochloropis gaditana. Nat Commun 3:686.

9. Gobler CJ, Berry DL, Dyhrman ST, Wilhelm SW, Salamov A, Lobanov AV, Zhang Y, Collier JL, Wurch LL, Kustka AB, Dill BD, Shah M, VerBerkmoes NC, Kuo A, Terry A, Pangilinan J, Lindquist EA, Lucas S, Paulsen IT, Hattenrath-Lehmann TK, Talmage SC, Walker EA, Koch F, Burson AM, Marcoval MA, Tang YZ, Lecleir GR, Coyne KJ, Berg GM, Bertrand EM, Saito MA, Gladyshev VN, Grigoriev IV. 2011. Niche of harmful alga Aureococcus anophagefferens revealed through ecogenomics. Proc Natl Acad Sci U S A 108:4352-7.

10. Links MG, Holub E, Jiang RH, Sharpe AG, Hegedus D, Beynon E, Sillito D, Clarke WE, Uzuhashi S, Borhan MH. 2011. De novo sequence assembly of Albugo candida reveals a small genome relative to other biotrophic oomycetes. BMC Genomics 12:503.

11. Kemen E, Gardiner A, Schultz-Larsen T, Kemen AC, Balmuth AL, Robert-Seilaniantz A, Bailey K, Holub E, Studholme DJ, Maclean D, Jones JD. 2011. Gene gain and loss during evolution of obligate parasitism in the white rust pathogen of Arabidopsis thaliana. PLoS Biol 9:e1001094.

12. Baxter L, Tripathy S, Ishaque N, Boot N, Cabral A, Kemen E, Thines M, Ah-Fong A, Anderson R, Badejoko W, Bittner-Eddy P, Boore JL, Chibucos MC, Coates M, Dehal P, Delehaunty K, Dong S, Downton P, Dumas B, Fabro G, Fronick C, Fuerstenberg SI, Fulton L, Gaulin E, Govers F, Hughes L, Humphray S, Jiang RH, Judelson H, Kamoun S, Kyung K, Meijer HJG, Minx P, Morris P, Nelson J, Phuntumart V, Qutob D, Rehmany A, Rougon-Cardoso A, Ryden P, Torto-Alalibo T, Studholme D, Wang Y, Win J, Wood J, Clifton SW, Rogers J, Van den Ackerveken G, Jones JD, McDowell JM, et al. 2010. Signatures of adaptation to obligate biotrophy in the Hyaloperonospora arabidopsidis genome. Science 330:1549-51.

13. Lamour KH, Mudge J, Gobena D, Hurtado-Gonzales OP, Schmutz J, Kuo A, Miller NA, Rice BJ, Raffaele S, Cano LM, Bharti AK, Donahoo RS, Finley S, Huitema E, Hulvey J, Platt D, Salamov A, Savidor A, Sharma R, Stam R, Storey D, Thines M, Win J, Haas BJ, Dinwiddie DL, Jenkins J, Knight JR, Affourtit JP, Han CS, Chertkov O, Lindquist EA, Detter C, Grigoriev IV, Kamoun S, Kingsmore SF. 2012. Genome sequencing and mapping reveal loss of heterozygosity as a mechanism for rapid adaptation in the vegetable pathogen Phytophthora capsici. Mol Plant Microbe Interact 25:1350-60.

14. Studholme DJ, McDougal RL, Sambles C, Hansen E, Hardy G, Grant M, Ganley RJ, Williams NM. 2016. Genome sequences of six Phytophthora species associated with forests in New Zealand. Genom Data 7:54-6.

15. Haas BJ, Kamoun S, Zody MC, Jiang RH, Handsaker RE, Cano LM, Grabherr M, Kodira CD, Raffaele S, Torto-Alalibo T, Bozkurt TO, Ah-Fong AM, Alvarado L, Anderson VL, Armstrong MR, Avrova A, Baxter L, Beynon J, Boevink PC, Bollmann SR, Bos JI, Bulone V, Cai G, Cakir C, Carrington JC, Chawner M, Conti L, Costanzo S, Ewan R, Fahlgren N, Fischbach MA, Fugelstad J, Gilroy EM, Gnerre S, Green PJ, Grenville-Briggs LJ, Griffith J, Grunwald NJ, Horn K, Horner NR, Hu CH, Huitema E, Jeong DH, Jones AM, Jones JD, Jones RW, Karlsson EK, Kunjeti SG, Lamour K, Liu Z, et al. 2009. Genome sequence and analysis of the Irish potato famine pathogen *Phytophthora infestans*. Nature 461:393-8.

16. Tyler BM, Tripathy S, Zhang X, Dehal P, Jiang RH, Aerts A, Arredondo FD, Baxter L, Bensasson D, Beynon JL, Chapman J, Damasceno CM, Dorrance AE, Dou D, Dickerman AW, Dubchak IL, Garbelotto M, Gijzen M, Gordon SG, Govers F, Grunwald NJ, Huang W, Ivors KL, Jones RW, Kamoun S, Krampis K, Lamour KH, Lee MK, McDonald WH, Medina M, Meijer HJG, Nordberg EK, Maclean DJ, Ospina-Giraldo MD, Morris PF, Phuntumart V, Putnam NH, Rash S, Rose JK, Sakihama Y, Salamov AA, Savidor A, Scheuring CF, Smith BM, Sobral BW, Terry A, Torto-Alalibo TA, Win J, Xu Z, Zhang H, et al. 2006. Phytophthora genome sequences uncover evolutionary origins and mechanisms of pathogenesis. Science 313:1261-6.

17. Sharma R, Xia X, Cano LM, Evangelisti E, Kemen E, Judelson H, Oome S, Sambles C, van den Hoogen DJ, Kitner M, Klein J, Meijer HJG, Spring O, Win J, Zipper R, Bode HB, Govers F, Kamoun S, Schornack S, Studholme DJ, Van den Ackerveken G, Thines M. 2015. Genome analyses of the sunflower pathogen *Plasmopara halstedii* provide insights into effector evolution in downy mildews and *Phytophthora*. BMC Genomics 16:741.

18. Adhikari BN, Hamilton JP, Zerillo MM, Tisserat N, Levesque CA, Buell CR. 2013. Comparative genomics reveals insight into virulence strategies of plant pathogenic oomycetes. PLoS One 8:e75072.

19. Jiang RH, de Bruijn I, Haas BJ, Belmonte R, Lobach L, Christie J, van den Ackerveken G, Bottin A, Bulone V, Diaz-Moreno SM, Dumas B, Fan L, Gaulin E, Govers F, Grenville-Briggs LJ, Horner NR, Levin JZ, Mammella M, Meijer HJG, Morris P, Nusbaum C, Oome S, Phillips AJ, van Rooyen D, Rzeszutek E, Saraiva M, Secombes CJ, Seidl MF, Snel B, Stassen JH, Sykes S, Tripathy S, van den Berg H, Vega-Arreguin JC, Wawra S, Young SK, Zeng Q, Dieguez-Uribeondo J, Russ C, Tyler BM, van West P. 2013. Distinctive expansion of potential virulence genes in the genome of the oomycete fish pathogen Saprolegnia parasitica. PLoS Genet 9:e1003272.

20. Hovde BT, Deodato CR, Hunsperger HM, Ryken SA, Yost W, Jha RK, Patterson J, Monnat RJ, Jr., Barlow SB, Starkenburg SR, Cattolico RA. 2015. Genome Sequence and Transcriptome Analyses of Chrysochromulina tobin: Metabolic Tools for Enhanced Algal Fitness in the Prominent Order Prymnesiales (Haptophyceae). PLoS Genet 11:e1005469.

21. Read BA, Kegel J, Klute MJ, Kuo A, Lefebvre SC, Maumus F, Mayer C, Miller J, Monier A, Salamov A, Young J, Aguilar M, Claverie JM, Frickenhaus S, Gonzalez K, Herman EK, Lin YC, Napier J, Ogata H, Sarno AF, Shmutz J, Schroeder D, de Vargas C, Verret F, von Dassow P, Valentin K, Van de Peer Y, Wheeler G, Emiliania huxleyi Annotation C, Dacks JB, Delwiche CF, Dyhrman ST, Glockner G, John U, Richards T, Worden AZ, Zhang X, Grigoriev IV. 2013. Pan genome of the phytoplankton *Emiliania* underpins its global distribution. Nature 499:209-13.

22. Srivastava M, Simakov O, Chapman J, Fahey B, Gauthier ME, Mitros T, Richards GS, Conaco C, Dacre M, Hellsten U, Larroux C, Putnam NH, Stanke M, Adamska M, Darling A, Degnan SM, Oakley TH, Plachetzki DC, Zhai Y, Adamski M, Calcino A, Cummins SF, Goodstein DM, Harris C, Jackson DJ, Leys SP, Shu S, Woodcroft BJ, Vervoort M, Kosik KS, Manning G, Degnan BM, Rokhsar DS. 2010. The Amphimedon queenslandica genome and the evolution of animal complexity. Nature 466:720-6.

23. Suga H, Chen Z, de Mendoza A, Sebe-Pedros A, Brown MW, Kramer E, Carr M, Kerner P, Vervoort M, Sanchez-Pons N, Torruella G, Derelle R, Manning G, Lang BF, Russ C, Haas BJ, Roger AJ, Nusbaum C, Ruiz-Trillo I. 2013. The Capsaspora genome reveals a complex unicellular prehistory of animals. Nat Commun 4:2325.

24. Fairclough SR, Chen Z, Kramer E, Zeng Q, Young S, Robertson HM, Begovic E, Richter DJ, Russ C, Westbrook MJ, Manning G, Lang BF, Haas B, Nusbaum C, King N. 2013. Premetazoan genome evolution and the regulation of cell differentiation in the choanoflagellate Salpingoeca rosetta. Genome Biol 14:R15.

25. King N, Westbrook MJ, Young SL, Kuo A, Abedin M, Chapman J, Fairclough S, Hellsten U, Isogai Y, Letunic I, Marr M, Pincus D, Putnam N, Rokas A, Wright KJ, Zuzow R, Dirks W, Good M, Goodstein D, Lemons D, Li W, Lyons JB, Morris A, Nichols S, Richter DJ, Salamov A, Sequencing JG, Bork P, Lim WA, Manning G, Miller WT, McGinnis W, Shapiro H, Tjian R, Grigoriev IV, Rokhsar D. 2008. The genome of the choanoflagellate Monosiga brevicollis and the origin of metazoans. Nature 451:783-8.

26. Urushihara H, Kuwayama H, Fukuhara K, Itoh T, Kagoshima H, Shin IT, Toyoda A, Ohishi K, Taniguchi T, Noguchi H, Kuroki Y, Hata T, Uchi K, Mohri K, King JS, Insall RH, Kohara Y, Fujiyama A. 2015. Comparative genome and transcriptome analyses of the social amoeba Acytostelium subglobosum that accomplishes multicellular development without germ-soma differentiation. BMC Genomics 16:80.

27. Eichinger L, Pachebat JA, Glockner G, Rajandream MA, Sucgang R, Berriman M, Song J, Olsen R, Szafranski K, Xu Q, Tunggal B, Kummerfeld S, Madera M, Konfortov BA, Rivero F, Bankier AT, Lehmann R, Hamlin N, Davies R, Gaudet P, Fey P, Pilcher K, Chen G, Saunders D, Sodergren E, Davis P, Kerhornou A, Nie X, Hall N, Anjard C, Hemphill L, Bason N, Farbrother P, Desany B, Just E, Morio T, Rost R, Churcher C, Cooper J, Haydock S, van Driessche N, Cronin A, Goodhead I, Muzny D, Mourier T, Pain A, Lu M, Harper D, Lindsay R, Hauser H, et al. 2005. The genome of the social amoeba Dictyostelium discoideum. Nature 435:43-57.

28. Heidel AJ, Lawal HM, Felder M, Schilde C, Helps NR, Tunggal B, Rivero F, John U, Schleicher M, Eichinger L, Platzer M, Noegel AA, Schaap P, Glockner G. 2011. Phylogeny-wide analysis of social amoeba genomes highlights ancient origins for complex intercellular communication. Genome Res 21:1882-91.

29. Sucgang R, Kuo A, Tian X, Salerno W, Parikh A, Feasley CL, Dalin E, Tu H, Huang E, Barry K, Lindquist E, Shapiro H, Bruce D, Schmutz J, Salamov A, Fey P, Gaudet P, Anjard C, Babu MM, Basu S, Bushmanova Y, van der Wel H, Katoh-Kurasawa M, Dinh C, Coutinho PM, Saito T, Elias M, Schaap P, Kay RR, Henrissat B, Eichinger L, Rivero F, Putnam NH, West CM, Loomis WF, Chisholm RL, Shaulsky G, Strassmann JE, Queller DC, Kuspa A, Grigoriev IV. 2011. Comparative genomics of the social amoebae Dictyostelium discoideum and Dictyostelium purpureum. Genome Biol 12:R20.

30. Clarke M, Lohan AJ, Liu B, Lagkouvardos I, Roy S, Zafar N, Bertelli C, Schilde C, Kianianmomeni A, Burglin TR, Frech C, Turcotte B, Kopec KO, Synnott JM, Choo C, Paponov I, Finkler A, Heng Tan CS, Hutchins AP, Weinmeier T, Rattei T, Chu JS, Gimenez G, Irimia M, Rigden DJ, Fitzpatrick DA, Lorenzo-Morales J, Bateman A, Chiu CH, Tang P, Hegemann P, Fromm H, Raoult D, Greub G, Miranda-Saavedra D, Chen N, Nash P, Ginger ML, Horn M, Schaap P, Caler L, Loftus BJ. 2013. Genome of Acanthamoeba castellanii highlights extensive lateral gene transfer and early evolution of tyrosine kinase signaling. Genome Biol 14:R11.

31. Glockner G, Hulsmann N, Schleicher M, Noegel AA, Eichinger L, Gallinger C, Pawlowski J, Sierra R, Euteneuer U, Pillet L, Moustafa A, Platzer M, Groth M, Szafranski K, Schliwa M. 2014. The genome of the foraminiferan Reticulomyxa filosa. Curr Biol 24:11-8.

32. Curtis BA, Tanifuji G, Burki F, Gruber A, Irimia M, Maruyama S, Arias MC, Ball SG, Gile GH, Hirakawa Y, Hopkins JF, Kuo A, Rensing SA, Schmutz J, Symeonidi A, Elias M, Eveleigh RJ, Herman EK, Klute MJ, Nakayama T, Obornik M, Reyes-Prieto A, Armbrust EV, Aves SJ, Beiko RG, Coutinho P, Dacks JB, Durnford DG, Fast NM, Green BR, Grisdale CJ, Hempel F, Henrissat B, Hoppner MP, Ishida K, Kim E, Koreny L, Kroth PG, Liu Y, Malik SB, Maier UG, McRose D, Mock T, Neilson JA, Onodera NT, Poole AM, Pritham EJ, Richards TA, Rocap G, Roy SW, et al. 2012. Algal genomes reveal evolutionary mosaicism and the fate of nucleomorphs. Nature 492:59-65.
