# Supplementary figures and images for: The Ancient Link between G-Protein-Coupled Receptors and C-Terminal Phospholipid Kinase Domains
Source: mBio. 2018 Jan 23;9(1):e02119-17. doi: 10.1128/mBio.02119-17 (PMC5784254; doi:10.1128/mBio.02119-17)

a

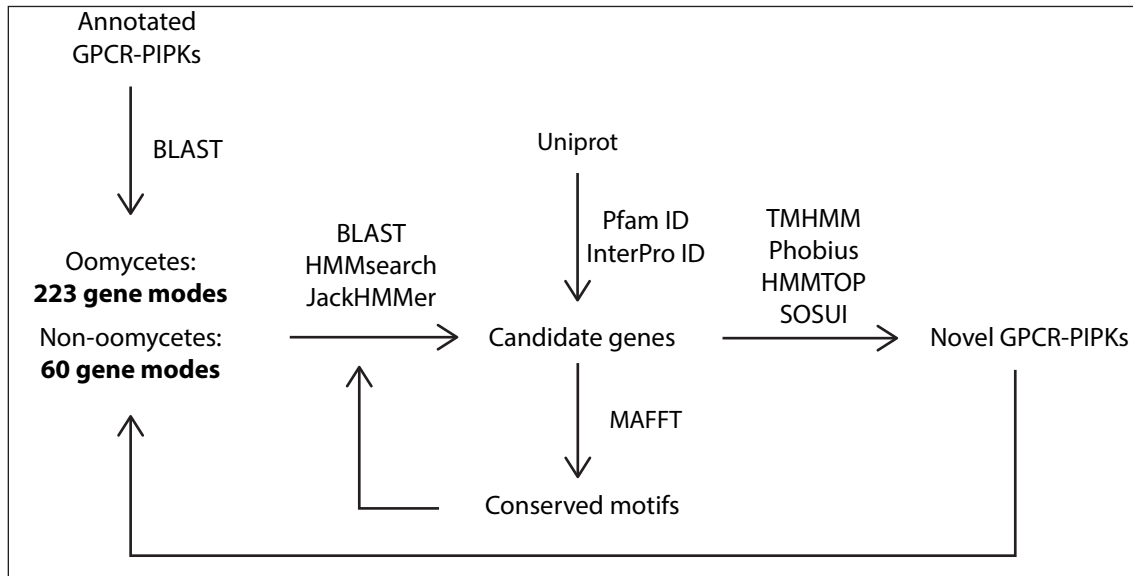

b

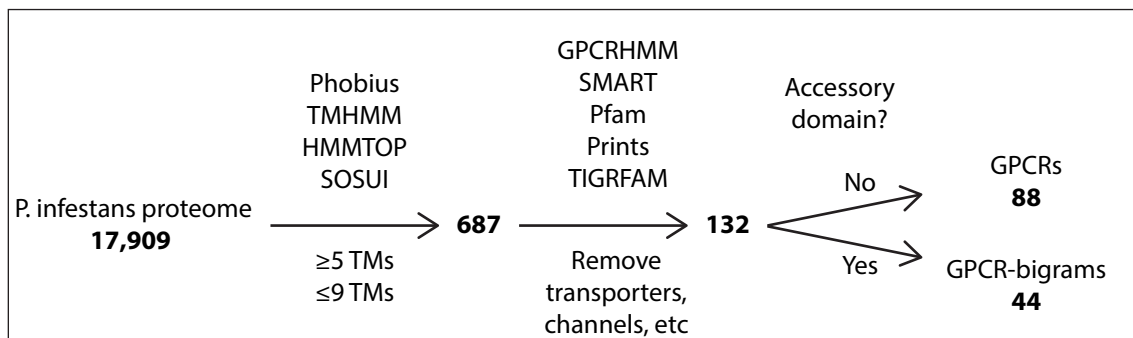

Supplement: FIG S1 [file mbo001183681sf1.pdf]

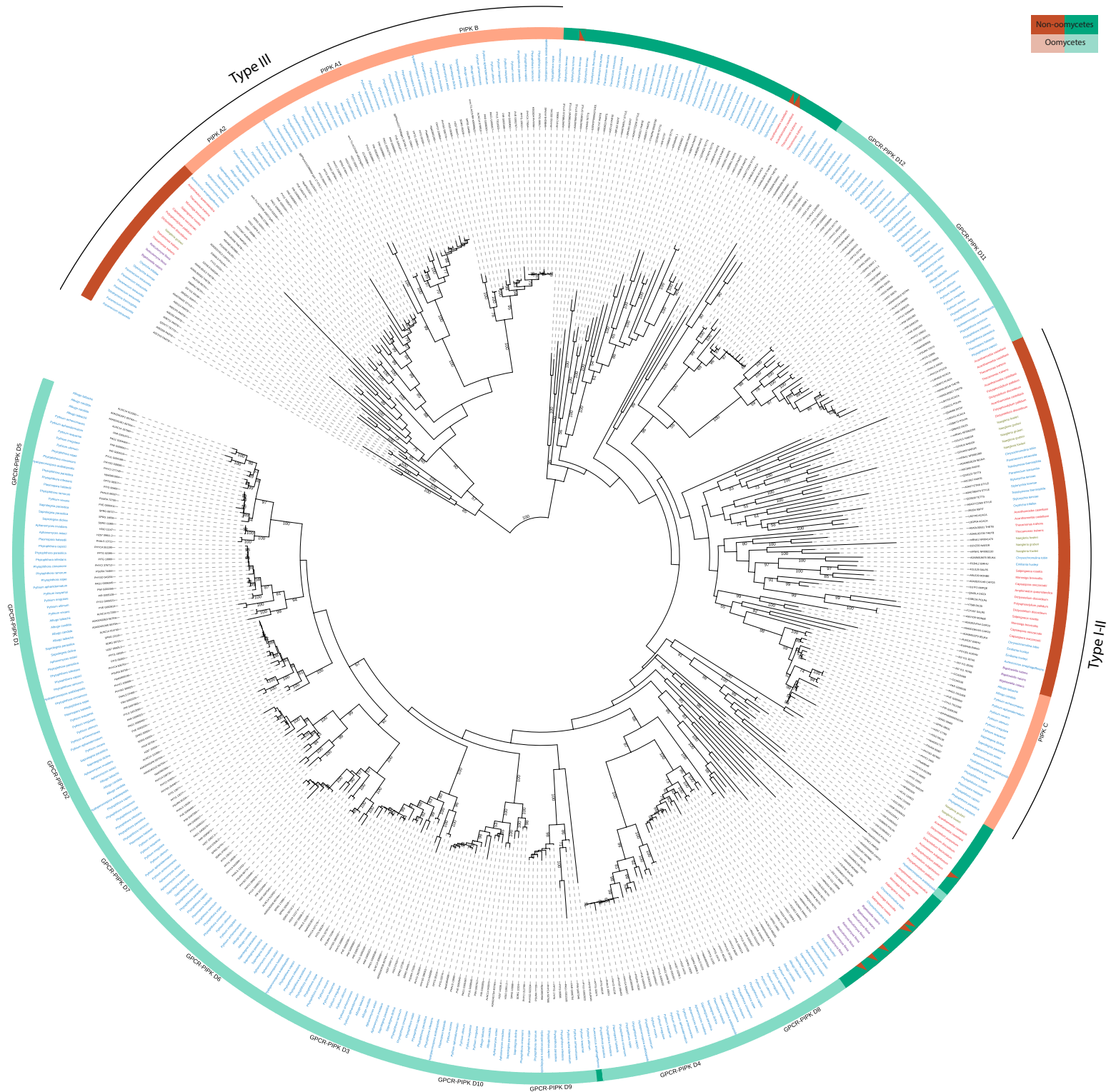

Supplement: FIG S2 [file mbo001183681sf2.pdf]

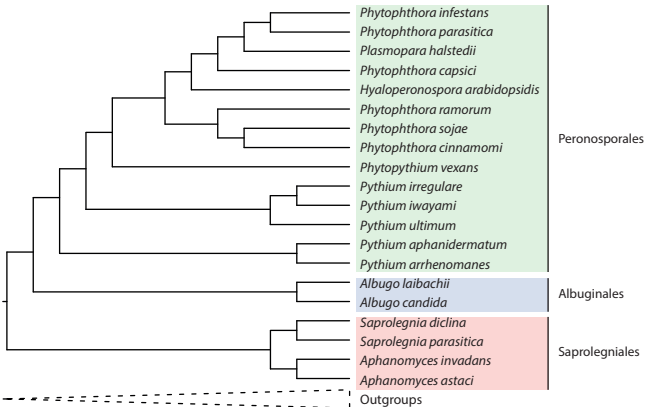

Supplement: FIG S3 [file mbo001183681sf3.pdf]
